# Metagenome Sequencing Reveals the Midgut Microbiota Makeup of *Culex pipiens quinquefasciatus* and Its Possible Relationship With Insecticide Resistance

**DOI:** 10.3389/fmicb.2021.625539

**Published:** 2021-02-25

**Authors:** Yi-ting Wang, Rui-xin Shen, Dan Xing, Chen-pei Zhao, He-ting Gao, Jia-hong Wu, Ning Zhang, Heng-duan Zhang, Yan Chen, Tong-yan Zhao, Chun-xiao Li

**Affiliations:** ^1^State Key Laboratory of Pathogen and Biosecurity, Beijing Key Laboratory of Vector Borne and Natural Focus Infectious Disease, Institute of Microbiology and Epidemiology, Beijing, China; ^2^School of Basic Medical Sciences, Guizhou Medical University, Guiyang, China; ^3^College of Life Sciences, Ludong University, Yantai, China; ^4^College of Life Science and Technology, Beijing University of Chemical Technology, Beijing, China

**Keywords:** *Culex pipiens quinquefasciatus*, metagenomics, gut microbiota, deltamethrin, resistance

## Abstract

Midgut microbiota can participate in the detoxification and metabolism processes in insects, but there are few reports on the relationship between midgut microbiota and insecticide resistance in mosquitoes. In this study, we performed metagenomic sequencing on a susceptible strain (SS), a field-collected Hainan strain (HN), and a deltamethrin-resistant strain (RR) of *Culex pipiens quinquefasciatus* to understand the diversity and functions of their midgut microbiota. The results revealed differences in midgut microbiota among the three strains of *Cx. pipiens quinquefasciatus*. At the phylum level, Proteobacteria was the most prominent, accounting for nearly 70% of their midgut microbes. At the genus level, *Aeromonas* made up the highest proportion. In addition, *Aeromonas*, *Morganella*, *Elizabethkingia*, *Enterobacter*, *Cedecea*, and *Thorsellia* showed significant differences between strains. At the species level, *Bacillus cereus*, *Enterobacter cloacae complex* sp. 4DZ3-17B2, *Streptomyces* sp. CNQ329, and some species of *Pseudomonas* and *Wolbachia* were more abundant in the two resistant strains. Principal component analysis (PCA) showed that the SS strain had significantly different metagenomic functions than the two deltamethrin-resistant strains (HN and RR strain). The HN and RR strains differed from the SS strain in more than 10 Kyoto Encyclopedia of Genes and Genomes (KEGG) pathways. The analysis of species abundance and functional diversity can provide directions for future studies.

## Introduction

Microorganisms are an important part of the ecosystem and are intimately involved in the physiology of plants, animals, and humans (Engel and Moran, 2013). Insects are the most diverse animal group known on earth. Insects can maintain a population advantage in a complex ecological environment and depends largely on the various symbiotic microorganisms in their bodies (Tang et al., 2012). The microorganisms in insects mainly reside in their midgut and include bacteria, fungi, viruses, and archaea. These symbiotic microorganisms directly or indirectly regulate the growth, reproduction, immune defense, and other physiological activities of insects (Crotti et al., 2012). The midgut microbiota is divided into autochthonous and allochthonous communities. The autochthonous communities are the main microbes involved in the physiological functions of insects.

Recently, studies have shown that there is a relationship between insect midgut symbiotic bacteria and its resistance to insecticides in insects (Xia et al., 2013; Kach et al., 2018). Midgut microbiota mediate resistance mainly in two ways. One is the direct metabolism of chemical insecticides by symbiotic bacteria. Kikuchi et al. (2012) found that *Burkholderia* mediated the resistance of *Riptortus pedestris* to an organophosphorus insecticide, fenitrothion, by directly metabolizing the insecticide. *Citrobacter* sp. CF-BD plays a key role in the degradation of trichlorfon by *Bactrocera dorsalis* (Cheng et al., 2017). In addition, midgut microbiota indirectly affect insect resistance to insecticides by increasing the activity of detoxification enzymes or enhancing their gene expression. Pang et al. (2018) found that the symbiotic bacterium *Arsenophonus* strain (S-type) enhanced the resistance of *Nilaparvata lugens* to imidacloprid by increasing this insect’s P450s enzyme activity and UGT gene expression.

*Culex pipiens quinquefasciatus* is an important mosquito vector throughout the world. This species can transmit a variety of vector-borne infectious diseases, including Bancroft’s filariasis and Japanese encephalitis, and is a potential vector for West Nile virus and Western equine encephalitis virus (Nuss et al., 2018; Xu et al., 2019). Controlling mosquitoes is the main way to prevent and control mosquito-borne infectious diseases (Liu, 2015). At present, chemical control is the most important measures for the prevention and control of *Cx. pipiens quinquefasciatus*. However, due to the long-term use of large quantities of chemical insecticides, the insecticide resistance of *Cx. pipiens quinquefasciatus* has become increasingly prominent (Reid et al., 2018), and the extensive use of insecticides has also had a great impact on the environment. Deltamethrin is the most widely used pyrethroid in the world. The resistance of mosquitoes to it has become a very serious problem (Liu, 2015).

Most previous studies on mosquito resistance to insecticides have focused on their insecticide resistance genes. In recent years, the relationship between the insecticide resistance of mosquitoes and their symbiotic bacteria has attracted increasing attention. Symbiotic bacteria can enhance the metabolic activity of *Anopheles stephensi* in response to organophosphate insecticides, thus promoting resistance to these insecticides (Soltani et al., 2017). Hong et al. (2014) found that the expression of *Wolbachia* was higher in the deltamethrin-resistant strains of *Cx. pipiens pallens*, showing that *Wolbachia* is related to deltamethrin resistance in mosquitoes. Dada et al. (2018) reported that in fenitrothion-resistant strains of *An. albimanus*, metagenomic sequencing revealed enrichment of *Klebsiella pneumoniae*. These results showed that there is a certain correlation between the midgut microbiota and the insecticide resistance of mosquitoes.

For many years, the only way to identify microorganisms in the natural environment was to separate and cultivate them with traditional methods. However, due to technical limitations, traditional methods of separation and culturing can no longer meet the requirements of current research on insect midgut microbiota. With the rapid development of high-throughput sequencing, metagenomic sequencing has shown great advantages in the study of midgut microbiota (Mou et al., 2008). Using metagenomic sequencing, we can analyze and study the population diversity, biological activity, and functional roles of midgut microbiota. In this study, a susceptible strain (SS), a field Hainan strain (HN), and a deltamethrin-resistant strain (RR) of *Cx. pipiens quinquefasciatus* were selected for metagenomic sequencing. The microbial diversity and metabolic functions of different strains were analyzed in order to clarify the relationship between the symbiotic midgut microbiota of *Cx. pipiens quinquefasciatus* and its resistance to deltamethrin and to provide a new target for the biological control of *Cx. pipiens quinquefasciatus*.

## Materials and Methods

### Mosquito Strains

Three strains of *Cx. pipiens quinquefasciatus* were used in this study. The SS strain is a laboratory strain, which was originally from Guangzhou and has been kept in the laboratory for more than 10 years without exposure to any insecticides. The Hainan strain (HN strain) was collected from Haikou City, Hainan Province, in 2013, and was reared in the laboratory until this study. The RR strain was obtained by exposing the HN strain to deltamethrin in the laboratory for 30 generations. The three strains have been kept in the same laboratory and given the same feeding regimes. The mosquitoes were reared at 26 ± 1°C, 75 ± 5% relative humidity, and a light/dark schedule of 14 h:10 h. The adult mosquitoes from the three strains were fed with 8% sugar water and 3–5 days after emergence, fed with blood meal to breed the next generation. Before metagenomic sequencing, the larvae of these three strains were bioassayed, and the LC_50_ value for deltamethrin of the SS, HN, and RR strains were 0.0000029, 0.014, and 0.572 μg/ml, respectively. The LC_50_ values of the HN strain and RR strain are much higher than that of SS strain (Shen et al., 2020).

### DNA Extraction of Mosquito Midgut

The midguts of the mosquitoes were removed from adult female *Cx. pipiens quinquefasciatus* at 3–5 days after emergence. Seventy female *Cx. pipiens quinquefasciatus* were rinsed with 75% ethanol for 90 s and then rinsed with sterile water three times. Each mosquito midgut was gently pulled out under a dissecting microscope, excess tissue was removed, and the midgut was rinsed with 1 × phosphate-buffered saline (PBS), placed in 100 μ l 1 × PBS, and frozen at −80°C. Three replicates were performed. The microbial genomic DNA of the sample was extracted with the QIAamp DNA Microbiome Kit (Shanghai, China). The concentration, total amount, purity, and degradation of the samples were tested, and the samples were then sent to Biomark Technologies (Beijing, China) for metagenomic sequencing.

### Library Construction and Sequencing

After the genomic DNA of the sample passed the quality test, the DNA was fragmented by mechanical shearing (ultrasonication). Then, the fragmented DNA was purified, end-repaired, 3′-adenylated, ligated to the sequencing adapters, and then electrophoresed in agarose gel to perform fragment size selection. PCR amplification was conducted to form a sequencing library. The constructed library was first subjected to library quality inspection, and the Illumina sequencing platform was used for metagenomic sequencing of the qualifying library.

### Bioinformatics Analysis

Quality control and host filtering were conducted on the original reads obtained by sequencing. After obtaining valid data (clean reads), the metagenome was assembled, and the unused reads from each sample were combined to form a mixed assembly to detect low-abundance species in the samples. Next, gene prediction, species annotation, and common-function database annotation were conducted. Taxonomic analysis was conducted using clean reads, and the species composition and abundance information of the samples were calculated.

### Statistical Analysis

The metagenomeSeq of R software (Segata et al., 2011) was used to screen different species at the genus and species level, the DESeq2 package in R software was used to screen different KEGG Orthology (KO) pathways, and we used | Log2FoldChange| ≥ 2, padj ≤ 0.05 as a condition to screen different species and KO pathways. Then, we used the R package pheatmap to draw the heat map of the KO pathways. According to the previous literature, we identified the species and KO pathways related to resistance. We used STAMP software to screen the different species and pathways according to the principal component analysis (PCA) method and Welch’s *t* test (two-side) method under the condition of *p* < 0.05. After picking out the relevant species and functional pathways, we drew the extended error bar diagram. SPSS Statistics 21 was used to perform ANOVA, Dunnett *t* test was used to analyze the abundance of microbiota, the *p* value was Bonferroni corrected, and *p* < 0.05 was considered statistically significant.

## Results

### Basic Statistics

Metagenomic sequencing of the SS, HN, and RR strains was conducted on the Illumina sequencing platform. After host sequence removal and quality control, the sequencing depths of nine samples were all approximately 10G. The number of reads of the nine samples was between 33,090,131 and 39,863,554. The Q20 values were all above 96%, the Q30 values were all above 90%, and the N50 values were all above 900 bp, indicating that the gene assembly had high integrity and good quality, ensuring the credibility of the subsequent analysis (Supplementary Table 1).

**FIGURE 1:**
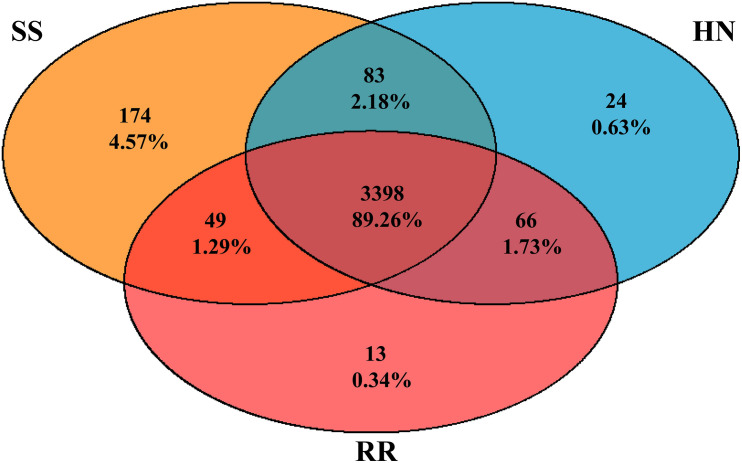
Venn diagram of the number of shared and unique bacteria genera among the microbiota of the SS strain (orange), the HN strain (blue), and the RR strain (red).

**FIGURE 2:**
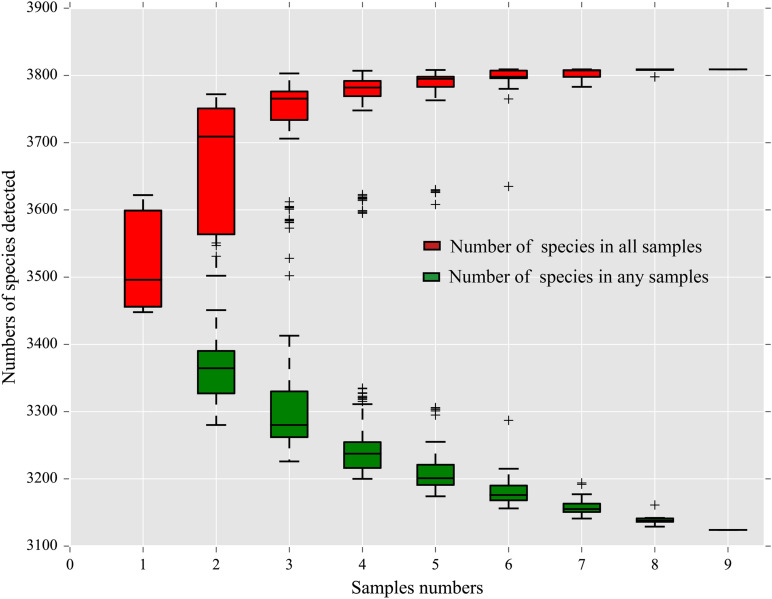
Box plot of species accumulation curves.

A total of 3,807 species, including 95 Archaea, 3,362 Bacteria, 1 Eukaryota, 780 Fungi, 4 Metazoa, and 157 Viruses (Supplementary Table 2), were annotated by the sequencing. Among them, 3,398 (89.26%) were shared by all three strains. There were 174, 24, and 13 species unique to the midguts of the SS, HN, and RR strain, respectively, accounting for 4.57, 0.63, and 0.34% of the total number of species, respectively. Sixty-six species were shared by the HN strain and the RR strain but not the SS strain, accounting for 1.73% of the total number of species (Figure 1). The species accumulation curve revealed that as the sample size increased, the curve rose sharply, indicating that many species were discovered, and then, the curve tended to be flattened, indicating that the number of species had reached saturation and that we had enough samples to cover most of the species (Figure 2).

### The Composition of Midgut Microbiota in *Cx. pipiens quinquefasciatus*

**FIGURE 3:**
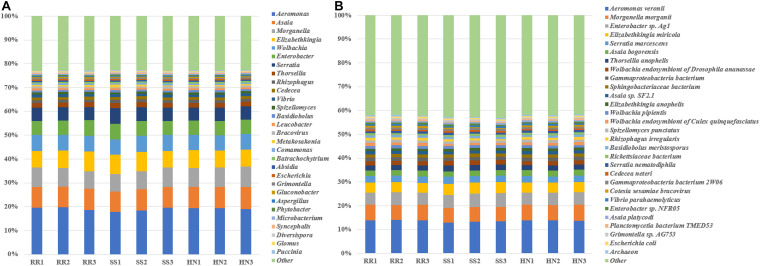
The taxonomic composition of the dominant microorganisms of the three strains of *Cx. pipiens quinquefasciatus*. **(A)** The average taxonomic composition of the dominant genera of the three strains of *Cx. pipiens quinquefasciatus.* **(B)** The average taxonomic composition of the dominant species of the three strains of *Cx. pipiens quinquefasciatus.*

At the phylum level (Supplementary Figure 1), the top 10 phyla accounted for more than 95% of all phyla, including five bacterial phyla (Proteobacteria, Bacteroidetes, Actinobacteria, Firmicutes, and Planctomycetes) and five fungal phyla (Mucoromycota, Ascomycota, Chytridiomycota, Basidiomycota, and Zoopagomycota). Among them, Proteobacteria predominated, accounting for nearly 70% of the microbiota phyla. There was no significant difference among strains at the phylum level. At the genus level, the top 30 genera accounted for more than 77%; among them, *Aeromonas* dominated, accounting for nearly 20%; the six genera *Asaia*, *Morganella*, *Elizabethkingia*, *Wolbachia*, *Enterobacter*, and *Serratia* each accounted for 5–10%; and the remaining genera each accounted for <2% (Figure 3A). At the species level, the top 30 species accounted for more than 55%, of which *Aeromonas veronii* had the highest abundance, accounting for nearly 14%; *Morganella morganii* and *Enterobacter* sp. Ag1 were also relatively rich, accounting for approximately 6 and 5%, respectively. The remaining species accounted for <5% (Figure 3B).

**FIGURE 4:**
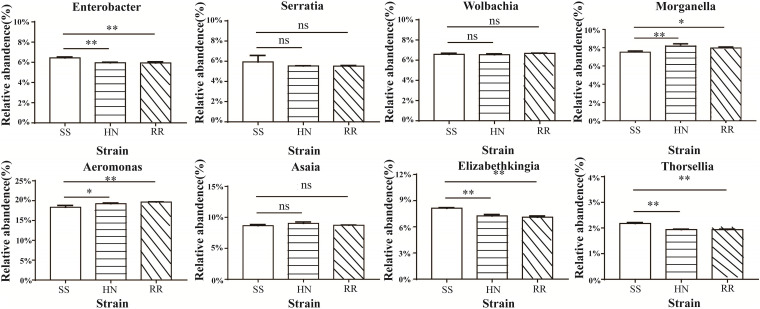
Histogram of midgut microbiota in the SS, HN, and RR strains of *Cx. pipiens quinquefasciatus* (*p < 0.05, **p < 0.01; ns, no significant difference).

At the genus level, we conducted a difference analysis for species with relatively high abundance; *Aeromonas* and *Morganella* were more abundant in the HN strain and the RR strain than in the SS strain, while *Elizabethkingia*, *Enterobacter*, and *Thorsellia* were significantly more abundant in the SS strain (*p* < 0.01). The levels of *Wolbachia*, *Serratia*, and *Asaia* in the three strains were not significantly different (Figure 4).

The metagenomeSeq tool was used for screening at the genus level. The results showed that the two deltamethrin-resistant strains (HN and RR) had eight microbial genera that were more abundant than they were in strain SS: *Acaricomes*, *Desertifilum*, *Claussenomyces*, *Oleiagrimonas*, *Citromicrobium*, *Mizugakiibacter*, *Aciduliprofundum*, and *Chitinimonas*. The HN and RR strains had 10 microbial genera with lower abundance than in the SS strain, including *Flexivirga*, *Thalassotalea*, *Porphyromonas*, *Phormidesmis*, *Methylomicrobium*, *Verminephrobacter*, *Basfia*, *Palleronia*, *Halogeometricum*, and *Candidatus Thioglobus* (Supplementary Table 3).

The metagenomeSeq tool was also used for screening at the species level, and it showed that strains HN and RR had 17 species with greater abundance than in the SS strain, including *Aciduliprofundum boonei*, *Desertifilum* sp. IPPAS B-1220, *Phycisphaerales bacterium*, *Phialocephala subalpina*, *Claussenomyces* sp., *Oleiagrimonas soli*, and *Arthrobacter luteolus*. The HN and RR strains had 31 microorganisms with lower abundance than in the SS strain, including *Citrobacter braakii*, *Citrobacter amalonaticus*, *Streptococcus mitis*, *Bacteroides thetaiotaomicron*, *Paenibacillus thiaminolyticus*, *Sphingomonas haloaromaticamans*, *Halogeometricum borinquense*, and *Proteus penneri* (Supplementary Table 4).

**FIGURE 5:**
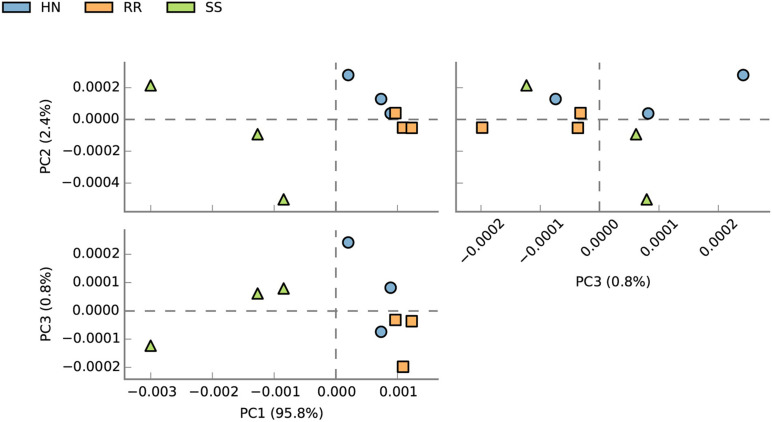
Principal component analysis (PCA) of the abundance of midgut microbial species in the SS, HN, and RR strains of *Cx. pipiens quinquefasciatus*.

**FIGURE 6:**
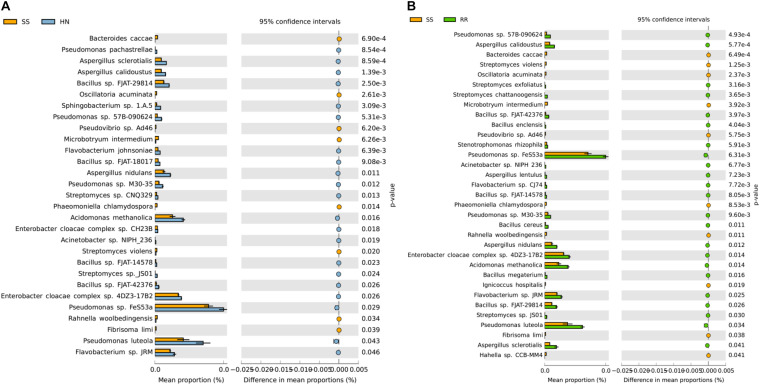
The differentially expressed bacteria screened by Welch’s *t* test. **(A)** Comparative analysis of the relative abundance of species between the SS strain and the HN strain. **(B)** Comparative analysis of the relative abundance of species between the SS strain and the RR strain.

At the species level, PCA was conducted for abundance analysis. The results showed that there were significant differences between the SS strain and the HN strain as well as between the SS strain and the RR strain (Figure 5). PCA1 explained 95.8% of the variance. Based on Welch’s *t* test, comparing the RR strain and the SS strain, a total of 1,172 species with significant differences were screened (*p* < 0.05). Among them, 598 strains were more abundant in the RR strain, and the remaining 574 strains were more abundant in the SS strain. Comparing the HN strain and the SS strain, a total of 1,007 strains with significant differences were screened (*p* < 0.05). Among them, 491 strains were more abundant in the HN strain, and the remaining 516 strains were more abundant in the SS strain. Among the above-mentioned different bacteria, we searched through the literature to screen out strains with large differences between the mosquito strains. The strains that were upregulated (*p* < 0.05) in the two resistant strains were *Pseudomonas* (*Pseudomonas* sp. 57B-090624, *Pseudomonas* sp. FeS53a, *Pseudomonas* sp. M30-35, *Pseudomonas luteoia*), *Aspergillus* (*Aspergillus calidoustus*, *Aspergillus nidulans*, *Aspergillus sclerotialis*, *Wolbachia* endosymbiont of *Culex quinquefasciatus*, *Streptomyces* sp. JS01, *Bacillus* sp. FJAT-42F376, *Bacillus* sp. 14578, *Bacillus* sp. FJAT-29814), *Flavobacterium* sp. JRM, *Acinetobacter* sp. NIPH 236, *E. cloacae complex* sp. 4DZ3-17B2, and *Acidomonas* (*Acidomonas methanolica*). The strains that were downregulated (*p* < 0.05) in the two resistant strains include *Bacteroides caccae*, *Streptomyces violens*, *Oscillatoria acuminate*, *Microbotryum intermedium*, *Pseudovibrio* sp. Ad46, *Phaeomoniella chlamydospora*, *Rahnella woolbedingensis*, and *Fibrisoma limi*. The strains only upregulated in the RR strain were *Aspergillus* (*Aspergillus lentulus*), *Wolbachia pipientis*, *Streptomyces exfoliates*, *Streptomyces chattanoogensis*, *Bacillus enclensis*, *B. cereus*, *Bacillus megaterium*, *Flavobacterium* sp. CJ74, *Citrobacter* sp. MH181794, and *Stenotrophomonas rhizophila*. The strains only upregulated in the HN strain were *P. pachastrellae*, *Streptomyces* sp. CNQ329, *Bacillus* sp. FJAT-18017, *Flavobacterium johnsoniae*, intestinal *Enterobacter cloacae* complex sp. CH23B, and *Sphingobacterium* sp. 1.A.5 (Figure 6).

### Species Random Forest Regression and Prediction Analysis

**FIGURE 7:**
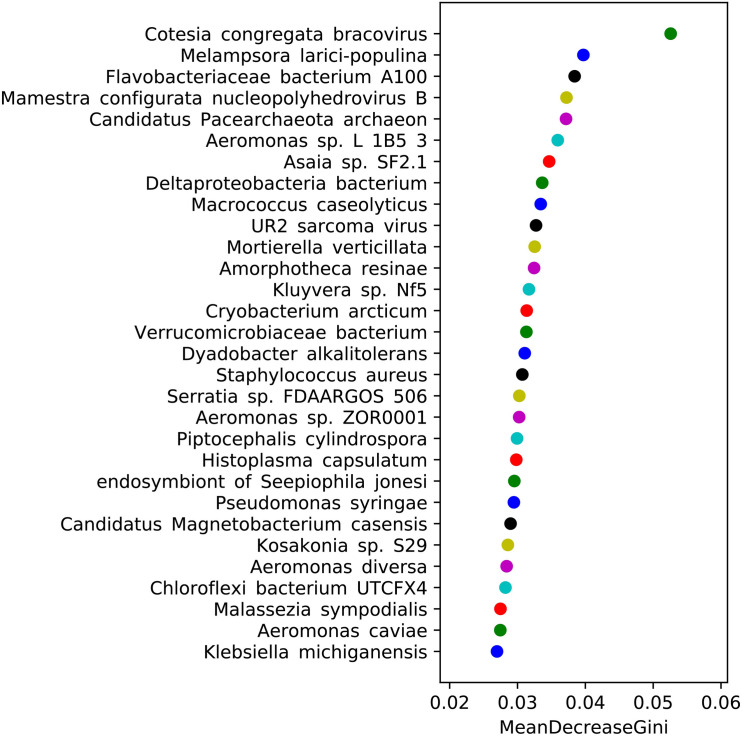
Species importance ranking map produced by random forest analysis. (The abscissa is the measure of species importance, and the ordinate is the species name sorted by importance).

Random forest analysis can efficiently and quickly select the most important species for classification (Figure 7). We selected the 30 most important species at the species level for analysis and found that the 10 most important species were *Cotesia congregata bracovirus*, *Melampsora larici-populina*, *F. bacterium A100*, *Mamestra configurata nucleopolyhedrovirus B*, *Candidatus* Pacearchaeota archaeon, *Aeromonas* sp. L1B53, *Asaia* sp. SF2.1, *Deltaproteobacteria bacterium*, *M. caseolyticus*, and UR2 sarcoma virus. By querying the correspondence between species and function, we found that the annotation results of *M. caseolyticus* were related to the functions of P450 enzymes and glutathione S-transferase. The annotation results of *Candidatus* Pacearchaeota archaeon and *Kluyvera* sp. Nf5 were related to the functions of sodium-dependent transport and insecticide metabolism enzymes, respectively. *F. bacterium A100* is a bacterium belonging to the genus *Flavobacteria*, and the specific function of the annotation result is not yet clear. *Serratia* sp. FDAARGOS506 and *P. syringae* belong to the genera *Serratia* and *Pseudomonas*, respectively, and were very important for the classification of the three strains. Other species may be involved in some life processes and may be related to the functions of enzymes involved in the life activities of mosquitoes, such as nucleic acid synthesis, molting, information processing, and synthesis of amino acids and proteins.

### Metagenomic Functional Analysis

Analysis of genes of the differentially expressed microbiota revealed that the annotated genes of microbiota with higher abundance in the deltamethrin-resistant strains primarily coded for enzymes involved in biological processes, such as bilirubin, nucleic acid, amino acid, and glyoxylic acid cycle processes and transmembrane protein synthesis. The upregulation of these enzymes increases the activity of these processes, which, in turn, provide more nutrients and energy to the mosquitoes. In addition, the annotation results of the microbiota with reduced abundance in the deltamethrin-resistant strains were related to the functions of proteins or enzymes such as ATP-binding proteins, ribosomal proteins, transfer proteins, transport proteins and transferases, ligases, phosphatases, oxidase, reductase, dehydrogenase, peptidase, and hydrolase (Supplementary Table 3).

**FIGURE 8:**
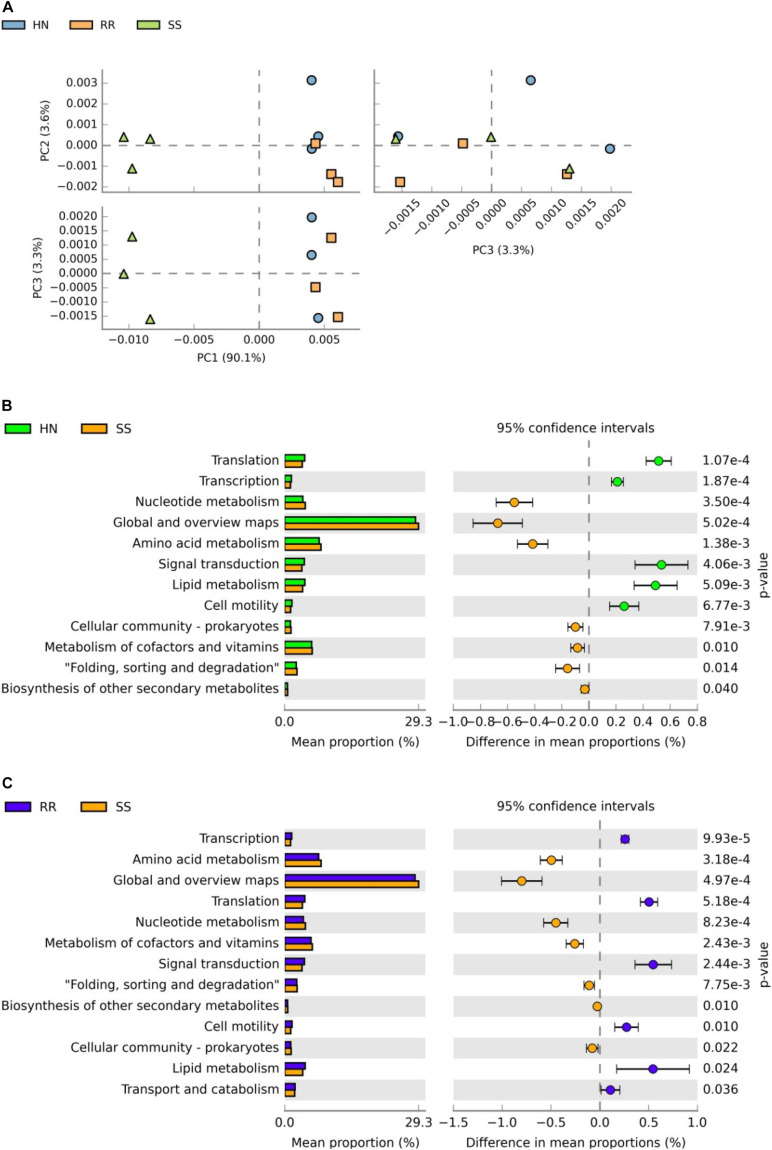
Analysis of differentially expressed Kyoto Encyclopedia of Genes and Genomes (KEGG) pathways. **(A)** Principal component analysis of KEGG pathways in the three mosquito strains. **(B)** Comparative analysis of the relative abundance of KEGG pathways between the SS strain and HN strain. **(C)** Comparative analysis of the relative abundance of KEGG pathways.

STAMP software was used to analyze the sequencing results, and the relative abundances of the Kyoto Encyclopedia of Genes and Genomes (KEGG) pathways related to midgut microbiota in the SS, HN, and RR strains were obtained (Supplementary Table 5). PCA showed that the SS strain had a different functional spectrum than the two deltamethrin-resistant strains: the SS strain had significant separation characteristics and a PCA1-explained variance of 90.1% (Figures 8A–C). Welch’s *t*-test (*p* < 0.05) was used to identify the KEGG pathways with significant differences between the two deltamethrin-resistant strains and the SS strain, including the metabolic and synthetic pathways involved in transcription, translation signal transduction, folding, classification, and cell motility. Among them, pathways related to substance synthesis, transportation, and catabolism were more abundant in the two deltamethrin-resistant strains, while pathways involved in the metabolism of amino acids, nucleotides, coenzymes, and vitamins were more abundant in the SS strain.

**FIGURE 9:**
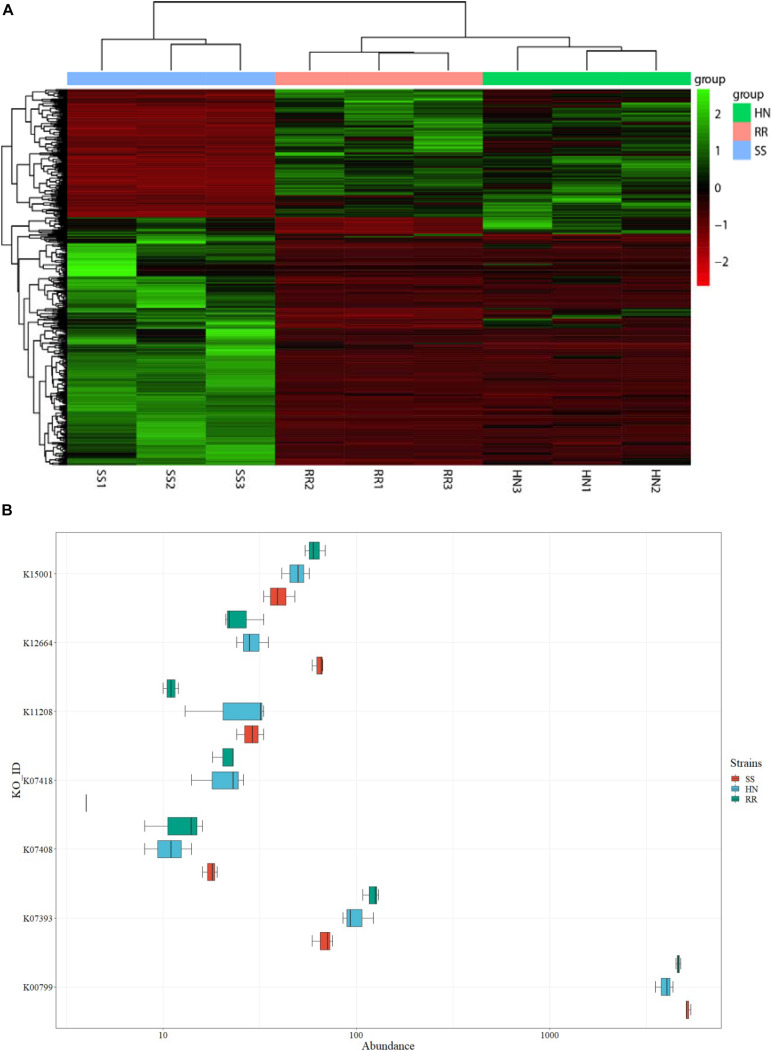
Analysis of differentially expressed Kyoto Encyclopedia of Genes and Genomes (KEGG) Orthology (KO) pathways in the three strains of *Cx. pipiens quinquefasciatus.* **(A)** KO pathway heat map of all differentially expressed microorganisms. **(B)** Box plot of differentially expressed KO pathways.

The DESeq2 package was used to screen and obtain the heat map of the differentially expressed KO pathways (Figure 9A and Supplementary Table 6). The heat map showed that the identified microbiota of each strain had good reproducibility, and color comparison showed that the two deltamethrin-resistant strains (HN and RR) were very different from the SS strain. Based on the detoxification enzymes related to deltamethrin resistance reported in the literature (Figure 9B), including glutathione S-transferase (K00799, K07393, and K11208) (Li et al., 2018) and cytochrome P450s (K07408, K07418, K12664, and K15001) (Chandor-Proust et al., 2013), we selected the different KO pathways. In the HN and RR strains, the KO pathways K07393, K07418, and K15001were more abundant than in the SS strain, putatively recognized as the glutathione S-transferase, cytochrome P450 family 2 subfamily J, and cytochrome P450 family pathways, respectively. Pathways K00799, K11208, K07408, and K12664 were less abundant in the HN and RR strains than in the SS strain, representing the glutathione S-transferase, GST-like protein, cytochrome P450 family 1 subfamily A polypeptide 1, and cytochrome P450 family 26 subfamily B pathway, respectively. K00799, K07393, K07418, and K12664 had significantly different abundances in the SS strain than in the two deltamethrin-resistant strains (*p* < 0.05), while the abundance of the K15001 pathway differed only between the SS and RR strains.

## Discussion

The composition of the mosquito midgut microbiota varies with the species, growth stage, and geographical area. Food and water are the main sources of midgut microbiota in mosquitoes and are also the main reasons for the differences in the composition of the midgut microbiota among different populations of mosquitoes. The three strains of *Cx. pipiens quinquefasciatus* in this study had been reared in the laboratory for many years; therefore, the consistency of their midgut microbiota was very high. In this situation, the microbiota identified by differential analysis would be more likely to be related to insecticide resistance.

Proteobacteria is the dominant phylum in *Cx. pipiens quinquefasciatus*, and Firmicutes is also found in *Cx. pipiens quinquefasciatus* (Rani et al., 2009; Gusmao et al., 2010; Boissiere et al., 2012). In this study, 76.4% of microorganisms sequenced by metagenomics were bacteria. Among them, Proteobacteria accounted for the largest proportion, at 70%, which is consistent with previous studies. The 10 genera accounting for the largest proportions of microorganisms in adult mosquitoes were *Aeromonas*, *Asaia*, *Morganella*, *Elizabethkingia*, *Wolbachia*, *Enterobacter*, *Serratia*, *Thorsellia*, *Rhizophagus*, and *Cedecea*. *Asaia* can synthesize vitamin B to provide nutrition for mosquitoes (Crotti et al., 2010; Chouaia et al., 2012), and *Wolbachia* can stimulate mosquitoes to produce reactive oxygen species to activate the immune system, leading to the production of antimicrobial peptides to fight viruses (Pan et al., 2012). The high levels of *Enterobacter* and *Serratia* are also notable. These genera can produce hemolytic enzymes and promote the digestion of food after blood sucking (Gusmao et al., 2010; Gaio Ade et al., 2011), and they play key roles in the growth, development, and life cycle of mosquitoes (Xia et al., 2013; Zhang et al., 2019).

Bacteria and fungi that play important roles in insecticide resistance in the life cycle of *Cx. pipiens quinquefasciatus* may be present throughout the lives of mosquitoes. A study of *Cx. pipiens quinquefasciatus* larvae found bacteria represented by *Bacillus* sp. and *Pseudomonas* sp. in the midgut as well as the fungus *Aspergillus* (*Aspergillus* sp.) and the actinomycete *Streptomyces* (*Streptomyces* sp.) (Vasanthi and Hoti, 1992). The above-mentioned bacteria and fungi were also found in the adult mosquitoes in the present study.

*B. cereus* (Zhang et al., 2016), *Acidomonas* sp. (Paingankar et al., 2005), *Streptomyces aureus* (Chen et al., 2011), *P. fluorescens*, *Enterobacter cloacae* (Hao et al., 2018), and *Aspergillus niger* (Liang et al., 2005) can degrade deltamethrin. Whether other species of *Bacillus*, *Acidomonas* sp., *Pseudomonas* sp., *Streptomyces*, *Enterobacter* sp., and *Aspergillus* play a role in metabolism needs to be investigated further. The metagenomic sequencing of this study found that *B. cereus* and *E. cloacae* complex sp. 4DZ3-17B2 in the RR strain was significantly more abundant than in the SS strain, indicating that they may metabolize deltamethrin in *Cx. pipiens quinquefasciatus*. The annotation result of *Streptomyces* sp. CNQ329 showed that it was a cytochrome P450 gene. P450s participate in the detoxification metabolism of deltamethrin. This study showed that the abundance of *Streptomyces* sp. CNQ329 in the HN strain was higher than that in the SS strain, suggesting that *Streptomyces* sp. CNQ329 may contribute to the resistance of *Cx. pipiens quinquefasciatus* to deltamethrin.

Tago et al. (2006) and Singh (2009) have shown that the application of fenitrothion sharply increases the abundance of *Pseudomonas* and *Flavobacterium*, which can degrade fenitrothion to 3-methyl-4-nitrophenol, a compound with no insecticide activity, and then further metabolize 3-methyl-4-nitrophenol into a carbon source for its growth (Kikuchi et al., 2012). Hong et al. (2014) found that *Wolbachia* was more abundant in deltamethrin-resistant strains of *Cx. pipiens pallens*, suggesting that *Wolbachia* contributes to deltamethrin resistance. *Pseudomonas* plays a role in the metabolism of organophosphates and pyrethroids (Zhang et al., 2018). This study showed that the abundance of a *Wolbachia* and four *Pseudomonas* strains in the two resistant mosquito strains was higher than that in the SS strains, and there were significant differences (*p* < 0.05), suggesting that the resistance of *Cx. pipiens quinquefasciatus* to deltamethrin may be closely related to the activity of *Pseudomonas* and *Wolbachia*. This finding shows that microorganisms not only have a metabolic effect on an insecticide and that the metabolism of the insecticides may furthermore require the synergistic effect of multiple microorganisms, and the development of insecticide resistance may also be the result of the synergistic effect of multiple mechanisms.

Random forest analysis helped us identify the 30 most important species in the *Cx. pipiens quinquefasciatus* midgut, whose corresponding annotated proteins were related to the classification of the three strains of mosquitoes. The three mosquito strains were basically the same except for their collection location and insecticide exposure conditions. Among these 30 species, *Flavobacteriaceae bacterium A100* is a bacterium belonging to the genus *Flavobacterium*. Flavobacteria can degrade organophosphorus insecticides (Kikuchi et al., 2012). The annotation results of differentially expressed genes of *M. caseolyticus* revealed P450s and glutathione S-transferase. These two detoxification enzymes are associated with deltamethrin resistance (Chandor-Proust et al., 2013; Li et al., 2018). Therefore, we speculate that *M. caseolyticus* may contribute to the resistance of mosquitoes to deltamethrin. The annotation result of *Candidatus* Pacearchaeota archaeon revealed a sodium-dependent transporter, which may be related to Na^+^ ion channels. The pyrethroid resistance of mosquitoes is related to the insensitivity of sodium ion channels (Narahashi, 1988). Therefore, *Candidatus* Pacearchaeota archaeon may also participate in the production of deltamethrin resistance. The annotation result of *Kluyvera* sp. Nf5 revealed an enzyme related to insecticide metabolism, which may be involved in the metabolic process of insecticides or other substances. *Serratia* sp. FDAARGOS506 and *P. syringae* were also very important for the classification of the three strains. *Serratia* sp. and *Pseudomonas* can degrade insecticides (Xia et al., 2018). We speculate that these two bacteria may be involved in the deltamethrin resistance of *Cx. pipiens quinquefasciatus*.

The upregulated and downregulated bacterial strains were screened using the metagenomeSeq tool. Among them, *A. boonei*, an upregulated strain, can utilize many enzymes encoding proteolytic or peptide hydrolysis activity and use peptides in their main metabolic pathway (Reysenbach and Flores, 2008). *Campoletis sonorensisichnovirus* can change the growth, development, and immunity of its host (Fath-Goodin et al., 2006). The function of *Phycisphaerales bacterium* is to participate in the nitrogen and carbon cycles (Fukunaga et al., 2010). *Mesorhizobium alhagi* can use mannitol as a carbon source to produce many extracellular polysaccharides, which can affect cellular Na^+^ content and antioxidase activity (Liu et al., 2017). Among the downregulated bacteria, *Kluyvera georgiana* is attractive for its biodegradation of acrylamide and other aliphatic amides in the environment (Thanyacharoen et al., 2012). *C. amalonaticus* can utilize multiple carbon sources and can use sucrose as a substrate to achieve high succinic acid production, indicating that reduced carbon substrates help maximize the redox potential (Amulya and Mohan, 2019). *B. thetaiotaomicron* digests a variety of complex carbohydrates and produces short-chain carbohydrates and organic acids that the host can absorb as energy sources (Porter et al., 2018). *Paenibacillus thiaminolyticus* can use phenol (Chandra et al., 2011), and *S. haloaromaticamans* can degrade *o*-phenol (Perruchon et al., 2017). The endosymbiotic bacterium *Buchnera aphidicola* is essential to the nutrient metabolism and normal development of the host (Nakabachi and Ishikawa, 1997). In general, these bacteria directly or indirectly provide the host with nitrogen sources, carbon sources, amino acids, vitamins, and energy and affect the host’s life activities.

After the analysis of differentially expressed KO pathways, the IDs were searched against the P450 database, and it was found that the K07408, K07418, K12664, and K15001 pathways were related to the CYP303, CYP18, CYP301, and CYP4 superfamilies, respectively. The CYP4 (K15001) superfamily is related to the development of deltamethrin resistance in mosquitoes (Chang et al., 2014). K15001 is only significantly different in the RR strain, which may be the reason why the RR strain is more resistant to deltamethrin than the HN strain. We speculate that the CYP4 superfamily of the midgut microbiota may play a role in the production of *Cx. pipiens quinquefasciatus* resistance to deltamethrin. Although the CYP303, CYP18, and CYP301 superfamilies have not been reported to be related to deltamethrin resistance, there were differences in the KO pathways between deltamethrin-resistant and susceptible strains, and attention should be given to these superfamilies in future research.

In this study, we identified mosquito midgut microbiota through metagenomic sequencing, annotated their functions, explored the relationships between some of the midgut microbiota and insecticide resistance, and identified some new bacteria, fungi, and viruses that may be related to this resistance. In future research, certain target midgut microbes should be cultivated or antibiotics could be applied to remove them from the mosquito midgut to further clarify their functions.

## Data Availability Statement

The data presented in the study are deposited in the NCBI BioProject repository, accession number PRJNA680753.

## Author Contributions

Y-tW, Rx-S, and DX conducted the analysis of the sequencing data. Y-tW and Rx-S wrote the manuscript. C-pZ participated in the analysis of processes and performed the software mapping work with H-tG. J-hW and H-dZ completed the manuscript and initial proofreading of the data. NZ participated in the preparation of the samples. YC, T-yZ, and C-xL reviewed and provided suggestions for the manuscript. All authors contributed to the writing and approved the final version of the manuscript.

## Conflict of Interest

The authors declare that the research was conducted in the absence of any commercial or financial relationships that could be construed as a potential conflict of interest.

## Supplementary Material

Supplementary Figure 1 The taxonomic composition of the dominant phyla of the three strains of *Cx. pipiens quinquefasciatus*.

Supplementary Table 1 Statistics of metagenomic sequencing data.

Supplementary Table 2 All microorganisms were annotated by the sequencing in *Cx. pipiens quinquefasciatus*.

Supplementary Table 3 Different bacteria at the genus level screened by metagenomeSeq method.

Supplementary Table 4 Different bacteria at the species level screened by metagenomeSeq method.

Supplementary Table 5 The differentially expressed KEGG pathways in the three strains of *Cx. pipiens quinquefasciatus*.

Supplementary Table 6 The differentially expressed KEGG Orthology (KO) pathways in the three strains of *Cx. pipiens quinquefasciatus.*
