# Supplementary figures and images for: Metagenome Sequencing Reveals the Midgut Microbiota Makeup of Culex pipiens quinquefasciatus and Its Possible Relationship With Insecticide Resistance
Source: Front Microbiol. 2021 Feb 25;12:625539. doi: 10.3389/fmicb.2021.625539 (PMC7948229; doi:10.3389/fmicb.2021.625539)

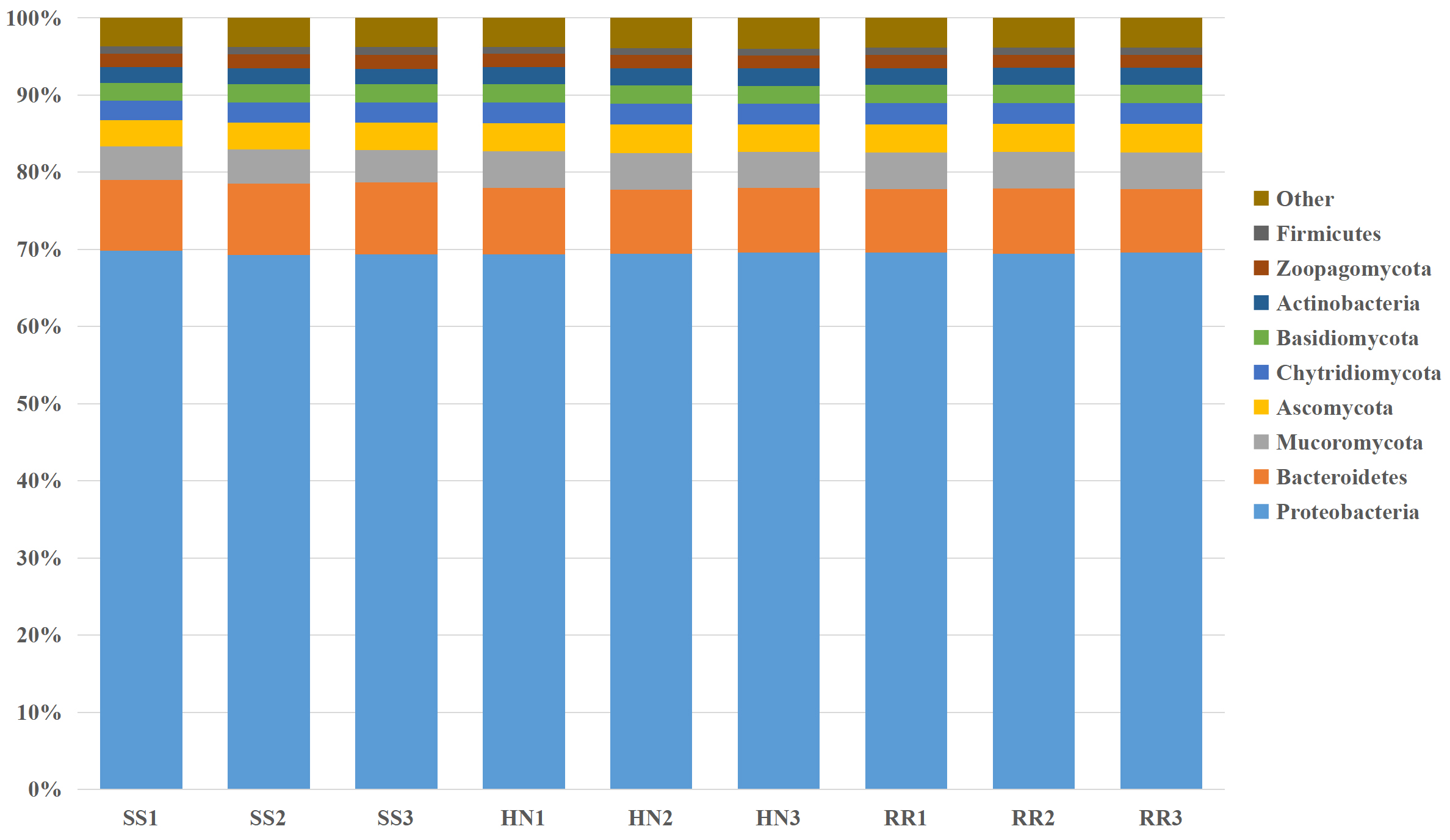

Supplement: Supplementary Figure 1 — The taxonomic composition of the dominant phyla of the three strains of Cx. pipiens quinquefasciatus. [file Image_1.JPEG]
