# Supplementary material for: Metagenome Sequencing Reveals the Midgut Microbiota Makeup of Culex pipiens quinquefasciatus and Its Possible Relationship With Insecticide Resistance
Source: Front Microbiol. 2021 Feb 25;12:625539. doi: 10.3389/fmicb.2021.625539 (PMC7948229; doi:10.3389/fmicb.2021.625539)
Supplement: Supplementary Table 1 — Statistics of metagenomic sequencing data. [file Table_1.DOCX]

**Table S1.** Statistics of metagenomic sequencing data

| **SampleID** | **UseData（G）** | **Raw data base（bp）** | **Clean data base（bp）** | **Number of Reads** | **GC（%）** | **Q20（%）** | **Q30（%）** | **N50（bp）** |
| --- | --- | --- | --- | --- | --- | --- | --- | --- |
| SS1 | 12.92072068 | 12686714146 | 10545989578 | 35239170 | 38.41 | 96.56 | 90.91 | 944 |
| SS2 | 13.70997672 | 13438235144 | 11110262874 | 37122086 | 37.55 | 96.64 | 91.07 | 946 |
| SS3 | 12.65363295 | 12413138644 | 10526608030 | 35171536 | 38.91 | 96.63 | 91.07 | 951 |
| HN1 | 13.70207058 | 13421197966 | 11235730958 | 37543083 | 38.29 | 96.58 | 90.98 | 1160 |
| HN2 | 14.60501102 | 14302646322 | 11930509546 | 39863554 | 37.48 | 96.58 | 90.91 | 1145 |
| HN3 | 12.0652012 | 11806944696 | 9904021224 | 33090131 | 37.68 | 96.68 | 91.12 | 1139 |
| RR1 | 13.08998474 | 12812337576 | 10799254286 | 36082837 | 38.03 | 96.67 | 91.08 | 1364 |
| RR2 | 13.20789225 | 12925851174 | 10846572488 | 36240944 | 38.57 | 96.63 | 91.05 | 1367 |
| RR3 | 14.27184421 | 13968106052 | 11411375130 | 38131497 | 37.57 | 96.54 | 90.88 | 1356 |
