# Supplementary material for: Metagenome Sequencing Reveals the Midgut Microbiota Makeup of Culex pipiens quinquefasciatus and Its Possible Relationship With Insecticide Resistance
Source: Front Microbiol. 2021 Feb 25;12:625539. doi: 10.3389/fmicb.2021.625539 (PMC7948229; doi:10.3389/fmicb.2021.625539)
Supplement: Supplementary Table 3 — Different bacteria at the genus level screened by metagenomeSeq method. [file Table_3.DOCX]

**Table S3.**  Different bacteria at the genus level screened by metagenomeSeq method

| **Differences species** | **Genus names** | **RR** | | **HN** | | **Annotation** |
| --- | --- | --- | --- | --- | --- | --- |
|  |  | **Fold Change** | **padj** | **Fold Change** | **padj** |  |
| Upregulated expression | Acaricomes | 6.32 | 6.56*10^-5^ | 6.08 | 2.33*10^-4^ | DUF3043 domain-containing protein |
|  | Desertifilum | 7.01 | 2.41*10^-6^ | 6.62 | 2.33*10^-5^ | heme oxygenase (biliverdin-producing) |
|  | Claussenomyces | 6.76 | 9.80*10^-6^ | 6.26 | 1.21*10^-4^ | DNA replication licensing factor, partial |
|  | Oleiagrimonas | 6.74 | 1.05*10^-5^ | 6.30 | 8.89*10^-5^ | DNA/RNA non-specific endonuclease |
|  | Citromicrobium | 6.59 | 1.65*10^-5^ | 6.94 | 3.77*10^-6^ | membrane dipeptidase |
|  | Mizugakiibacter | 6.32 | 6.59*10^-5^ | 6.16 | 1.67*10^-4^ | malate synthase A |
|  | Aciduliprofundum | 6.11 | 1.78*10^-4^ | 6.71 | 1.43*10^-5^ | serpin family protein |
|  | Chitinimonas | 2.20 | 4.40*10^-5^ | 2.48 | 1.54*10^-6^ | sodium:proton antiporter |
| Downregulated expression | Flexivirga | 2.55 | 1.18*10^-14^ | 2.54 | 9.54*10^-14^ | TP-binding protein |
|  | Thalassotalea | 5.97 | 4.34*10^-4^ | 5.93 | 6.52*10^-4^ | 50S ribosomal protein L3 N(5)-glutamine methyltransferase |
|  | Porphyromonas | 6.00 | 3.26*10^-4^ | 5.96 | 4.72*10^-4^ | alkaline phosphatase family protein |
|  | Phormidesmis | 6.05 | 3.02*10^-4^ | 6.02 | 4.09*10^-4^ | short-chain dehydrogenase |
|  | Methylomicrobium | 6.61 | 3.68*10^-5^ | 6.57 | 5.09*10^-5^ | conjugal transfer protein TraV |
|  | Verminephrobacter | 6.63 | 1.65*10^-5^ | 6.59 | 2.33*10^-5^ | M24 family metallopeptidase |
|  | Basfia | 6.68 | 1.48*10^-5^ | 6.64 | 2.02*10^-5^ | 3-deoxy-manno-octulosonate cytidylyltransferase |
|  | Palleronia | 6.74 | 1.05*10^-5^ | 6.70 | 1.43*10^-5^ | alkaline phosphatase |
|  | Halogeometricum | 6.74 | 1.05*10^-5^ | 6.70 | 1.43*10^-5^ | nucleoside hydrolase |
|  | Candidatus Thioglobus | 6.97 | 6.17*10^-6^ | 6.93 | 7.68*10^-6^ | choline dehydrogenase |
