# Supplementary material for: Metagenome Sequencing Reveals the Midgut Microbiota Makeup of Culex pipiens quinquefasciatus and Its Possible Relationship With Insecticide Resistance
Source: Front Microbiol. 2021 Feb 25;12:625539. doi: 10.3389/fmicb.2021.625539 (PMC7948229; doi:10.3389/fmicb.2021.625539)
Supplement: Supplementary Table 4 — Different bacteria at the species level screened by metagenomeSeq method. [file Table_4.DOCX]

**Table S4.** Different bacteria at the species level screened by metagenomeSeq method

| **Differences species** | **Species** | **RR strain** | | **HN strain** | | **Annotation** |
| --- | --- | --- | --- | --- | --- | --- |
|  |  | **Fold Change** | **padj** | **Fold**  **Change** | **padj** |  |
| Up-regulated | *Aciduliprofundum boonei* | 6.10 | 1.72×10^-4^ | 6.71 | 2.70×10^-5^ | serpin family protein |
|  | *Desertifilum sp. IPPAS B-1220* | 6.99 | 2.62×10^-6^ | 6.62 | 4.16×10^-5^ | heme oxygenase (biliverdin-producing) |
|  | *Phycisphaerales bacterium* | 6.96 | 4.47×10^-6^ | 7.23 | 1.35×10^-6^ | hypothetical protein C4547_03145 |
|  | *Phialocephala subalpina* | 6.76 | 8.21×10^-6^ | 6.22 | 1.93×10^-4^ | probable SWI1 Component of SWI/SNF global transcription activator complex |
|  | *Claussenomyces* sp*.* | 6.74 | 9.94×10^-6^ | 6.26 | 1.96×10^-4^ | DNA replication licensing factor, partial |
|  | *Oleiagrimonas soli* | 6.73 | 1.20×10^-5^ | 6.30 | 1.52×10^-4^ | DNA/RNA nonspecific endonuclease |
|  | *Arthrobacter luteolus* | 6.69 | 1.50×10^-5^ | 6.64 | 3.17×10^-5^ | NAD-dependent epimerase/dehydratase family protein |
|  | *Campoletis sonorensis ichnovirus* | 6.65 | 1.52×10^-5^ | 6.52 | 4.99×10^-5^ | innexin Vnx-g1 |
|  | *Actinomyces sp. Marseille-P3109* | 6.65 | 1.41×10^-5^ | 6.86 | 1.12×10^-5^ | amino acid adenylation domain-containing protein |
|  | *Streptomyces bottropensis* | 6.60 | 1.99×10^-5^ | 6.73 | 2.62×10^-5^ | trypsin-like serine protease |
|  | *Citromicrobium sp. JLT1363* | 6.58 | 1.88×10^-5^ | 6.94 | 7.22×10^-6^ | membrane dipeptidase |
|  | *SAR202 cluster bacterium Ae2-Chloro-G2* | 6.55 | 2.23×10^-5^ | 6.34 | 1.29×10^-4^ | hypothetical protein BZY65_02685 |
|  | *Mesorhizobium alhagi* | 6.53 | 2.40×10^-5^ | 6.19 | 2.34×10^-4^ | 5&apos;-nucleotidase domain-containing protein |
|  | *Mizugakiibacter sediminis* | 6.30 | 7.59×10^-5^ | 6.16 | 2.66×10^-4^ | malate synthase A |
|  | *Bacterium CG10 46 32* | 4.80 | 1.25×10^-8^ | 4.01 | 4.40×10^-5^ | hypothetical protein BK004_04600 |
|  | *Vibrio sp. E4404* | 3.33 | 1.12×10^-4^ | 4.38 | 4.36×10^-9^ | STAS domain-containing protein |
|  | *Herbiconiux ginsengi* | 3.19 | 4.03×10^-5^ | 3.32 | 4.71×10^-5^ | long-chain fatty acid--CoA ligase |
| Downregulated | *Citrobacter braakii* | -2.07 | 4.36×10^-5^ | -2.01 | 2.51×10^-4^ | type VI secretion system ATPase TssH |
|  | *Vibrio maritimus* | -2.28 | 1.56×10^-7^ | -2.73 | 1.19×10^-8^ | 3-dehydroquinate synthase |
|  | *Kluyvera georgiana* | -2.30 | 7.96×10^-5^ | -2.71 | 5.10×10^-5^ | MFS transporter |
|  | *Flexivirga sp. BO-16* | -2.57 | 9.62×10^-5^ | -2.54 | 4.95×10^-13^ | ATP-binding protein |
|  | *Legionella wadsworthii* | -2.98 | 1.12×10^-4^ | -5.27 | 1.66×10^-4^ | STAS domain-containing protein |
|  | *Vibrio xiamenensis* | -3.11 | 5.08×10^-7^ | -2.24 | 2.42×10^-4^ | 2-C-methyl-D-erythritol 4-phosphate cytidylyltransferase |
|  | *Pantoea sp. PSNIH6* | -3.97 | 2.99×10^-5^ | -3.23 | 2.79×10^-4^ | bifunctional biotin--[acetyl-CoA-carboxylase] ligase/biotin operon repressor BirA |
|  | *Citrobacter amalonaticus* | -4.19 | 8.13×10^-6^ | -4.85 | 3.55×10^-5^ | multifunctional transcriptional regulator/nicotinamide-nucleotide adenylyltransferase/ribosylnicotinamide kinase NadR |
|  | *Streptococcus mitis* | -6.18 | 1.39×10^-4^ | -6.13 | 3.32×10^-4^ | hypothetical protein SMIM3I_02223 |
|  | *Polyporus cuticulatus* | -6.26 | 1.02×10^-4^ | -6.20 | 2.37×10^-4^ | beta-tubulin, partial |
|  | *Bacteroides thetaiotaomicron* | -6.29 | 9.01×10^-5^ | -6.23 | 2.12×10^-4^ | hypothetical protein |
|  | *Paenibacillus thiaminolyticus* | -6.29 | 8.91×10^-5^ | -6.24 | 2.09×10^-4^ | NAD(P)/FAD-dependent oxidoreductase |
|  | *Sphingomonas haloaromaticamans* | -6.29 | 8.61×10^-5^ | -6.24 | 1.99×10^-4^ | cupin domain-containing protein |
|  | *Pseudoalteromonas sp. P1-30* | -6.32 | 8.29×10^-5^ | -6.27 | 1.93×10^-4^ | cytochrome c551 |
|  | *Rhabdoviridae environmental sample* | -6.33 | 8.23×10^-5^ | -6.27 | 1.92×10^-4^ | nucleocapsid protein |
|  | *Photobacterium sanctipauli* | -6.43 | 5.81×10^-5^ | -6.38 | 1.37×10^-4^ | DeoR family transcriptional regulator |
|  | *Buchnera aphidicola* | -6.44 | 4.89×10^-5^ | -6.39 | 1.21×10^-4^ | molecular chaperone DnaK |
|  | *Janthinobacterium agaricidamnosum* | -6.49 | 3.93×10^-5^ | -6.43 | 9.76×10^-5^ | chemotaxis protein |
|  | *Mesotoga sp. TolDC* | -6.51 | 3.88×10^-5^ | -6.46 | 9.64×10^-5^ | amino acid ABC transporter permease |
|  | *Pseudomonadales bacterium* | -6.53 | 2.89×10^-5^ | -6.47 | 7.05×10^-5^ | hypothetical protein EX270_06330 |
|  | *Methylomicrobium agile* | -6.63 | 3.63×10^-5^ | -6.58 | 8.82×10^-5^ | conjugal transfer protein TraV |
|  | *Verminephrobacter aporrectodeae* | -6.65 | 1.66×10^-5^ | -6.59 | 4.12×10^-5^ | M24 family metallopeptidase |
|  | *[Mannheimia] succiniciproducens* | -6.69 | 1.50×10^-5^ | -6.64 | 3.60×10^-5^ | 3-deoxy-manno-octulosonate cytidylyltransferase |
|  | *Serratia sp. HMSC15F11* | -6.70 | 1.38×10^-5^ | -6.64 | 3.36×10^-5^ | tRNA 2-selenouridine(34) synthase MnmH |
|  | *Capnocytophaga sputigena* | -6.72 | 1.50×10^-5^ | -6.67 | 3.60×10^-5^ | hypothetical protein |
|  | *Paenibacillus lentus* | -6.72 | 1.21×10^-5^ | -6.67 | 2.96×10^-5^ | hemolysin D |
|  | *Palleronia marisminoris* | -6.75 | 1.08×10^-5^ | -6.70 | 2.67×10^-5^ | alkaline phosphatase |
|  | *Halogeometricum borinquense* | -6.76 | 1.06×10^-5^ | -6.71 | 2.62×10^-5^ | nucleoside hydrolase |
|  | *Proteus penneri* | -6.82 | 8.88×10^-6^ | -6.77 | 2.26×10^-5^ | ribosomal protein L35 |
|  | *Candidatus Thioglobus sp.* | -6.98 | 5.96×10^-6^ | -6.93 | 1.40×10^-5^ | choline dehydrogenase |
|  | *Pantoea sp. B40* | -7.30 | 6.48×10^-7^ | -7.25 | 1.53×10^-6^ | MFS transporter |
